# Supplementary material for: The Efficacy and Safety of Prostaglandin E1 in the Management of Ischemic Retinal and Optic Nerve Diseases: A Systematic Review
Source: Neuroophthalmology. 2025 Aug 7;50(2):107–17. doi: 10.1080/01658107.2025.2544329 (PMC12990942; doi:10.1080/01658107.2025.2544329)
Supplement: Supplementary.docx [file IOPH_A_2544329_SM1630.docx]

**Supplemental Appendix**

**Table A.** The detailed search strategy.

| No. | Search Query |
| --- | --- |
| #1 | (prostaglandin* OR PG OR PGs OR PGE1 OR prostanoid* OR eicosanoid* OR Alprostadil OR Epoprostenol OR Dinoprostone ) |
| #2 | (CRAO OR BRAO OR “central retinal artery” OR “branch retinal artery” OR “cilioretinal artery” OR “cilioretinal arteries” OR “Arteria centralis retinae” OR “Ocular artery” OR “Ocular arteries” OR “Ocular blood vessel” OR “Ocular blood vessels” OR “ophthalmic artery” OR “Ocular stroke” OR “eye stroke” ) |
| #3 | #1 AND #2 |

**Table B.** The quality assessment of the included case report studies.

| Study | Q1 | Q2 | Q3 | Q4 | Q5 | Q6 | Q7 | Q8 |
| --- | --- | --- | --- | --- | --- | --- | --- | --- |
| Steigerwalt et al ,2017^23^ | Yes | Yes | Yes | Yes | Yes | Yes | No | Yes |
| Ikeda et al ,2004^13^ | Yes | Yes | Yes | Yes | Yes | Yes | Yes | Yes |
| Steigerwalt et al ,2011^24^ | Yes | Yes | Yes | Yes | Yes | Yes | Yes | Yes |
| Steigerwalt et al ,2011^12^ | Yes | Yes | Yes | Yes | Yes | Yes | No | Yes |
| Steigerwalt et al ,2016^22^ | Yes | Yes | Yes | Yes | Yes | Yes | No | Yes |
| Steigerwalt et al ,2003^17^ | Yes | Yes | Yes | Yes | Yes | Yes | No | Yes |
| Steigerwalt et al ,2020^21^ | Yes | Yes | Yes | Yes | Yes | Yes | No | Yes |

Q1: Were patient’s demographic characteristics clearly described?

Q2: Was the patient’s history clearly described and presented as a timeline?

Q3: Was the current clinical condition of the patient on presentation clearly described?

Q4: Were diagnostic tests or assessment methods and the results clearly described?

Q5: Was the intervention(s) or treatment procedure(s) clearly described?

Q6: Was the post-intervention clinical condition clearly described?

Q7: .Were adverse events (harms) or unanticipated events identified and described?

Q8: Does the case report provide takeaway lessons?

**Table C.** The quality assessment of the included case series studies.

| Study | Q1 | Q2 | Q3 | Q4 | Q5 | Q6 | Q7 | Q8 | Q9 | Q10 |
| --- | --- | --- | --- | --- | --- | --- | --- | --- | --- | --- |
| Chacko et al ,2023^14^ | Yes | Yes | Yes | Unclear | Unclear | Yes | Yes | Yes | No | Unclear |
| Steigerwalt et al ,2010^25^ | Yes | Yes | Yes | Unclear | Unclear | Yes | Yes | Yes | Yes | Unclear |
| Malbin et al ,2019^10^ | Yes | Yes | Yes | Yes | Unclear | Yes | Yes | Yes | Yes | Yes |
| Steigerwalt et al ,2009^26^ | Yes | Yes | Yes | Unclear | Unclear | Yes | Yes | Yes | No | Yes |
| Takai et al ,2013^15^ | Yes | Yes | Yes | Unclear | Unclear | Yes | Yes | Yes | Unclear | Yes |

Q1: Were there clear criteria for inclusion in the case series?

Q2: Was the condition measured in a standard, reliable way for all participants included in the case series?

Q3: Were valid methods used for identification of the condition for all participants included in the case series?

Q4: Did the case series have consecutive inclusion of participants?

Q5: Did the case series have complete inclusion of participants?

Q6: Was there clear reporting of the demographics of the participants in the study?

Q7: . Was there clear reporting of clinical information of the participants?

Q8 Were the outcomes or follow up results of cases clearly reported?

Q9: Was there clear reporting of the presenting site(s)/clinic(s) demographic information?

Q10: Was statistical analysis appropriate?
