# Supplementary material for: The Efficacy and Safety of Prostaglandin E1 in the Management of Ischemic Retinal and Optic Nerve Diseases: A Systematic Review
Source: Neuroophthalmology. 2025 Aug 7;50(2):107–17. doi: 10.1080/01658107.2025.2544329 (PMC12990942; doi:10.1080/01658107.2025.2544329)
Supplement: Manuscript_tracked.docx [file IOPH_A_2544329_SM1628.docx]

**The Efficacy and Safety of Prostaglandin E1 in the Management of Ischemic Retinal and Optic Nerve Diseases: A Systematic Review**

Hashem Abu Serhan^1*^, Ahmed Oweidah^2^, Abdulla Shaheen^3^, Laith O. Emoush^1^, Abdullah Bin Mahmoud^4^, Fatma Kassem Mohamed^1^, Usman Naeem^1^, Sara Irshaidat^5^, Ayman G Elnahry^5,6^

1. Department of Ophthalmology, Hamad Medical Corporation, Doha, Qatar; [HAbuserhan@hamad.qa](mailto:HAbuserhan@hamad.qa), [LEmoush@hamad.qa](mailto:LEmoush@hamad.qa), [Fmohamed15@hamad.qa](mailto:Fmohamed15@hamad.qa), [Unaeem@hamad.qa](mailto:Unaeem@hamad.qa)
2. Faculty of Medicine, Tanta University, Tanta, Egypt; [ahmed.oweidah.1@gmail.com](mailto:ahmed.oweidah.1@gmail.com)
3. Department of Ophthalmology, Bascom Palmer Eye Institute, University of Miami, Miami, FL, USA; [abdulla.shaheen.md@gmail.com](mailto:abdulla.shaheen.md@gmail.com)
4. Faculty of Medicine, Tripoli University, Tripoli, Libya; [abdullahbinmahmoud2001@gmail.com](mailto:abdullahbinmahmoud2001@gmail.com)
5. Sidra Medicine, Doha-Qatar; [SIrshaidat@sidra.org](mailto:SIrshaidat@sidra.org)
6. BayCare Clinic Eye Specialists, Aurora BayCare Medical Center, Green Bay, Wisconsin, USA; [ayman_elnahri@hotmail.com](mailto:ayman_elnahri@hotmail.com)
7. Department of Ophthalmology, Faculty of Medicine, Cairo University, Cairo, Egypt

***Corresponding author:**

Hashem Abu Serhan

Hamad Medical Corporation, Doha-Qatar

PO Box: 3050

Email: [HAbuserhan@hamad.qa](mailto:HAbuserhan@hamad.qa)

**Running title:** PGE1 & Retinal and Optic Nerve Ischemia**.**

**Conflict of Interest:** None declared.

**Abstract**

**Purpose:** To evaluate the efficacy and safety of intravenous prostaglandin E1 (PGE1) for the management of ischemic retinal and optic nerve diseases.

**Methods:** Our protocol was registered prospectively on PROSPERO (CRD42024524166). We searched 4 electronic databases [PubMed, Scopus, Web of Science, and Google Scholar] to retrieve all studies reported using PGE1 for patients with including CRAO, BRAO, A-AION, A-PION, ischemia in high myopia, NA-PION, and PAMM. We conducted a Wilcoxon signed-rank test to assess the effect of PGE1 on the visual acuity (VA) of included subjects. VA change was utilized to assess the degree of VA improvement. We assessed the quality of the included studies using the JBI tool.

**Results:** We included a total of 12 studies with a total of 30 cases. Age ranged from 40 to 97 years with a mean of 66.77 years for 15 females and 15 males. The most presented disease was CRAO (60%), followed by BRAO (10%), ischemia with high myopia (10%), non-arteritic posterior ischemic optic neuropathy (10%), arteritic posterior ischemic optic neuropathy (3.3%), arteritic anterior ischemic optic neuropathy (3.3%) and PAMM (3.3%). Time from symptoms onset to presentation was reported only in 25 cases (83.3%) with a median of 8 hours. Wilcoxon signed-rank test revealed significant improvement in visual acuity after IV PGE1 treatment (V = 465, p < 0.05).

**Conclusions:** Our analysis revealed preliminary evidence suggesting that IV PGE1 may be a potentially safe and effective treatment for improving VA in patients with posterior segment ischemia.

**Keywords:** Central retinal artery occlusion; branch retinal artery occlusion; ischemic optic neuropathy; ocular ischemia; prostaglandin; PGE1; myopia

**Introduction**

The posterior segment of the eye, comprising the retina, choroid, optic nerve head, and vitreous humor, plays a crucial role in visual information processing and transmission. This segment depends on a detailed vascular network stemming from the ophthalmic artery, with the central retinal artery supplying the inner retina and the ciliary arteries, including the short posterior ciliary arteries, supporting the optic nerve, retina, and choroid. Consequently, ischemic diseases such as retinal artery occlusion (RAO) and ischemic optic neuropathies (IONs) significantly impair visual function due to these tissues’ sensitivity to ischemia.

Treatment for ischemic optic neuropathies (IONs), including anterior ischemic optic neuropathy (AION) and posterior ischemic optic neuropathy (PION), varies depending on whether the condition is arteritic or non-arteritic and often involves corticosteroids, despite potential risks.^1^ For RAO, existing treatments aim to dislodge the clot to a more distal location through conservative measures such as ocular massage and paracentesis, or thrombolytics to dissolve the clots by intravenous (IV) or intra-arterial tissue plasminogen activator (tPA).^2-7^ However, both approaches are limited in efficacy, and the latter is associated with adverse effects, including intracerebral hemorrhage.^2-7^ Despite these efforts, current treatments often fall short of effectiveness in many cases, underscoring the need for different therapeutic options.

Prostaglandin E1 (PGE1), known for its wide-ranging applications in medical fields such as the treatment of erectile dysfunction^8^ and management of patent ductus arteriosus in neonates,^9^ acts through cyclic adenosine monophosphate mediated vasodilation and antiplatelet effects.^8,9^ This pharmacological profile has led multiple investigators to explore its use in vascular ophthalmic diseases of the retina and optic nerve. Despite the promising theoretical foundation for the use of PGE1 in ischemic ophthalmologic conditions, the empirical evidence remains sparse. To date, most insights into PGE1 efficacy and safety for retinal and optic nerve ischemia are derived from a limited number of case reports and small case series.^10-17^ This paucity of systematic research underscores a need for a comprehensive literature evaluation of PGE1 as a therapeutic option for posterior segment ischemia.

This systematic review seeks to synthesize existing evidence on the efficacy and safety of PGE1 in the management of ischemic diseases of the retina and optic nerve, with the goal of encouraging further investigation into its potential for treating ocular ischemia in the posterior segment.

**2. Materials and Methods**

**2.1. Study protocol and database search**

We conducted our review according to the Preferred Reporting for Systematic Review and Meta-Analysis (PRISMA) guidelines. We registered our protocol on PROSPERO (CRD42024524166). Our study did not require institutional review board (IRB) approval because there were no human subjects involved. We searched 4 electronic databases [PubMed, Scopus, Web of Science, and Google Scholar] to retrieve all studies that reported using intravenous prostaglandin E1 in the treatment of patients with CRAO or BRAO using the following keywords: “(prostaglandin* OR PG OR PGs OR PGE1 OR prostanoid* OR eicosanoid* OR Alprostadil OR Epoprostenol OR Dinoprostone)” AND (CRAO OR BRAO OR “central retinal artery” OR “branch retinal artery” OR “cilioretinal artery” OR “cilioretinal arteries” OR “Arteria centralis retinae” OR “Ocular artery” OR “Ocular arteries” OR “Ocular blood vessel” OR “Ocular blood vessels” OR “ophthalmic artery” OR “Ocular stroke” OR “eye stroke” OR “ischemic optic neuropathy” OR “ocular ischemia” OR “NAION” OR “AION” OR “PION”). We added Medical Subject Headings (MESH) terms to retrieve all relevant studies based on their indexed terms in included databases. In addition, after finishing the screening process, we conducted a manual search of references to identify any relevant studies that we could not identify through the original database search. The last search was done on 21 March 2024. Notably, we retrieved and screened only the first 200 papers from Google Scholar in accordance to the latest recommendations^18^. Our search strategy for each database is available in **Supplemental** **Table A.**

**2.2. Eligibility criteria**

We formulated our eligibility criteria according to the PICO framework^19^: participants were patients diagnosed with retinal or optic nerve diseases such as CRAO/BRAO, ischemic optic neuropathy, or ocular ischemia, the intervention was PGE1, the comparison was not limited, and the main outcome was the change in VA. No restrictions were made based on language, publication date, or study design. The secondary outcomes included the safety endpoints. Studies were included if they: (1) reported visual acuity before and after treatment. Meanwhile, we excluded studies if they (1) did not report VA before and after treatment or (2) did not have a full text.

**2.3. Screening and study selection**

We imported retrieved records from electronic databases in Endnote Software for duplicate removal. Then, we exported the citations as an Excel Sheet for screening. We performed the screening on two steps: title/abstract and full-text screening. Two reviewers [L.O.E., A.B.M.] carried out the screening process. Differences between reviewers were solved by a thorough discussion, and when necessary, the senior authors [H.A.S., A.G.E.] were consulted to give a final decision in unsolved disputes.

**2.4. Data extraction**

Two reviewers [A.O., H.A.S] developed the data extraction sheet with the use of Microsoft Excel (Microsoft, USA). This sheet consisted of Four parts. The first part included the baseline characteristics of included studies [title, authors’ names, year of publication, country, and study design] and of patients [age, gender, ethnicity]. The second part included clinical characteristics of cases [affected eye side, ocular history, general history and comorbidities, symptoms at presentation, diagnosis, and time from symptoms onset to treatment initiation]. The third part included data on PGE1 treatment [initial dose, frequency per day, duration of infusion, duration of treatment, cumulative dose, and adjuvant/additional treatment and reported adverse effects related to treatment]. The fourth part included VA data [visual acuity just before treatment (VA1), visual acuity after treatment (VA2) and time from treatment initiation to VA2 recording]. After extraction, we converted all visual acuity data into logMAR. Two reviewers [L.O.E., A.B.M.] extracted relevant data from finally included articles. Finally, two senior authors checked the accuracy of extracted data before the analysis [H.A.S., A.G.E.]. In addition, we assessed the methodological quality of the included studies using the Joanna Briggs Institute (JBI) critical appraisal tool for case reports and series studies^20^. The assessment is based on 8-10 domains; each is given a score of 0 (for no, not applicable, or not reported) or 1 (for yes). (**Supplemental** **Table B & C)**

**2.5. Data synthesis and statistical analysis**

Only studies that reported visual acuity before and after treatment were included in the analysis. All extracted visual acuity data were converted into logMAR. We conducted a wilcoxon signed-rank test using R studio software (version 2024.04.2), as the VA data (VA1 and VA2) did not follow a normal distribution, to assess the effect of PGE1 therapy on final VA among patients diagnosed with ischemic retinal and optic nerve diseases. The dataset comprised paired observations of VA before and after treatment from 30 cases. We used VA change (VA2 - VA1) to determine the extent of VA improvement.

**3. Results**

**3.1. Search results**

The results of the database search and screening are shown in **Figure 1**. The initial database search yielded 436 articles, of which 72 duplicates were removed through EndNote. Following the screening of 364 articles, the full texts of 18 articles were retrieved for full-text screening, of which 7 articles were excluded. Also, a manual search of references yielded one article^21^. So, only 12 articles were included in our review.

**3.2. Baseline characteristics of included studies and subjects**

A total of 12 studies were both qualitatively and quantitatively analyzed, out of which 7 were case reports^12,13,17,21-24^ and 5 were case series^10,14,15,25,26^ with a total of 30 cases. Studies were conducted in 3 countries: Italy (8 studies)^12,17,21-26^, Japan (2 studies)^13,15^ and USA (2 studies)^10,14^. (**Table 1)** Age ranged from 40 to 97 years with a mean of 66.77 years for 15 females and 15 males. (**Table 5**)

**3.3. Clinical characteristics of subjects**

Most cases presented with involvement of the left eye (63.3%). The most frequently reported disease was central retinal artery occlusion (CRAO) (n=18, 60%). Other reported diseases were branch retinal artery occlusion (BRAO) (n=3, 10%), ischemia with high myopia (n=3, 10%), non-arteritic posterior ischemic optic neuropathy (NA-PION) (n=3, 10%), arteritic posterior ischemic optic neuropathy (APION) (n=1, 3.3%), arteritic anterior ischemic optic neuropathy (AAION) (n=1, 3.3%) and paracentral acute middle maculopathy (PAMM) (n=1, 3.3%). Time from symptoms onset to presentation or treatment initiation was reported only in 25 cases (83.3%) with a median of 8 hours. (**Tables 2 and 5**)

**3.4. PGE1 treatment characteristics**

The median for the initial dose was 40 μg, for frequency of dose per day was 2, for duration of treatment was 4 days, and for cumulative dose until VA2 recording was 320 μg. In 4 cases, a steroid drug was given along with PGE1 (oral prednisone for 2 cases^22,24^ and IV 6- methylprednisolone for 2 cases^12,25^). Only one study reported using ocular massage^13^. Three studies reported that none of the cases experienced any adverse effects^10,13,24^. No major adverse events were reported in all studies. All cases received PGE1 intravenously except one case that received PGE1 as a topical skin cream applied to the inner surface of the forearm because IV PGE1 was not available^21^. Furthermore, another case received PGE1 as skin cream every 2 weeks in addition to initial IV PGE1^23^. **(Tables 3 and 5)**

**3.5. Patient’s characteristics regarding visual acuity**

All cases showed improvement in VA after treatment with PGE1 with 5 cases in 5 studies showing nearly improvement to the maximal visual acuity (0 logMAR)^15,17,21,22,24^**.** (**Table 4**) The median for VA in the affected eye just before treatment initiation (VA1) in logMAR was 2.30, for VA after treatment at first recorded follow-up time was 0.55 logMAR, for these follow-up times, was 7 days, and for change in VA (VA2-VA1) was -1.00 logMAR. (**Table 5)** Cases that were followed up again after VA2 recording showed either constant visual acuity or further improvement over VA2. No major adverse events were reported in all included subjects.

**3.6. Wilcoxon signed-rank test**

Wilcoxon signed-rank test revealed significant improvement in visual acuity after IV PGE1 treatment (V = 465, p < 0.05). This indicates that the median difference in VA before and after treatment was statistically significant.

**3.7. Quality assessment of the studies**

We assessed the quality of the included studies using the JBI checklist tool. All included studies used an appropriate population to test the PGE1 effect and clearly described patient characteristics. In addition, PGE1 administration techniques and outcome measurements were reported clearly. (**Supplemental** **Table B & C)**

**4.0 Discussion**

We observed a potential therapeutic role for IV PGE1 in improving VA in in eyes with various ischemic retinal and optic nerve diseases, including CRAO, BRAO, A-AION, A-PION, ischemia in high myopia, and NA-PION treated with PGE1. No severe drug-related adverse events were reported in the included cases.

Current treatment options for ischemic retinal and optic nerve disorders offer limited and often inconsistent efficacy and are associated with non-negligible risks. Steroid therapy efficacy in treating IONs appears variable, showing benefits in non-arteritic conditions but limited effectiveness in arteritic and surgical cases.^27^ This dichotomy is further illustrated by the case of relapsing NA-PION, where PGE1 proved effective after steroids failed to improve recurrent vision loss.^22^ This patient experienced recurrent vision loss without signs of inflammation, suggesting a different underlying disease mechanism,^22^ with PGE1 potential for enhancing blood flow or providing neuroprotection to the ischemic optic nerve. Also, steroids' inconsistent efficacy across ION types and the risk of serious complications underscores the need for alternative treatments. While this suggests a possible alternative pathophysiological mechanism responsive to vasodilatory therapy, it remains an isolated observation and should be interpreted cautiously.

Unlike corticosteroids, PGE1 exerts its effects by promoting vasodilation and inhibiting platelet aggregation, mechanisms that directly address the vascular compromise underlying posterior segment ischemia. Evidence from peripheral vascular disease has shown that a single intravenous dose of PGE1 can enhance blood flow for up to four weeks. ^28^ This might also explain the diminishing risk of subsequent vascular incidents, reinforcing IV PGE1 as a promising alternative or adjunct to existing treatments. Although extrapolation to the ocular circulation remains speculative, this pharmacologic profile supports further investigation in ophthalmic cases. Sobol K. et al investigated the intraarterial tPA in CRAO patients and found that there was a statistically significant improvement in VA, with a mean change of −0.76 (SD 0.91; range −2.4 to 0.85) logMAR (p=0.006).^29^ However, this procedure requires specialized expertise and equipment which may not be available in all centers. Furthermore, the potential of delivering PGE1 through a skin cream makes it a simple and noninvasive form of treatment that does not require sophisticated training or equipment.^21,23^ The relative simplicity of PGE1 administration—particularly in comparison to intra-arterial thrombolysis—may have cost advantages, although formal cost-effectiveness analyses are needed to substantiate this.

The timing of therapeutic interventions is critical in managing posterior segment ischemic diseases. In the cases included in our review, PGE1 demonstrated potential therapeutic benefit even when administered beyond the narrow time frame typically required for thrombolytic agents. Specifically, the median time to PGE1 initiation was approximately 8 hours, exceeding the 4.5-hour window generally accepted for tissue plasminogen activator (tPA) administration.^30^ The drug's rapid oxidation in the pulmonary circulation and the subsequent urinary excretion of metabolites within approximately 24 hours facilitate dosage adjustments tailored to individual patient responses.^31^ Lastly, evidence from animal studies indicates that PGE1 might exert genetic transcriptional effects that confer protection against ischemia.^32^ These various clinical properties support further investigation of PGE1 as a candidate therapy for posterior segment ischemia.

Our findings coincide with Suzuki T et al who investigated the anatomical and functional changes in 21 patients with CRAO treated with 10 µg/day intravenous liposomal PGE1. They showed that BCVA was significantly improved at 1 month and 3 months after the initial visit (from 2.18±0.60 to 1.54±0.84 and 1.53±0.88, p=0.030 and p=0.027, respectively) and no severe adverse effects were observed. ^11^We did not include Suzuki T et al study in our analysis because individual patient-level data were not reported.^11^ IV steroids were reported also in the management of BRAO in addition to IV PGE1 in the Steigerwalt D. et al report.^12^ They suggested that steroids could reduce ischemia–reperfusion injury. These multimodal approaches underscore the need for standardized treatment protocols in future research.

Definitive conclusions regarding PGE1 efficacy cannot be drawn from our findings. The results of our review should be approached with caution given that all included studies were case reports or small case series, without control groups or randomization, and inherently subject to reporting and publication bias. The retrospective design and selective outcome reporting further limit the reliability of the observed effects. Significant heterogeneity was present across the included cases, including differences in underlying diagnoses (e.g., CRAO, BRAO, NA-PION), PGE1 dosing regimens, routes of administration (intravenous vs. topical), adjunctive therapies (e.g., steroids), and follow-up durations. Moreover, diagnostic criteria were not consistently or uniformly reported, adding further variability. In addition, one major limitation is the natural history of CRAO, which does have some improvement of visual acuity in eyes with CF vision or worse. The estimate is about 10% for non-arteritic CRAO eyes.^33^ Some eyes with CRAO have improvements in vision even without treatment. In the absence of comparator arms, the true contribution of PGE1 to visual improvement cannot be isolated. Additionally, most cases had short follow-up durations, with a median of 8 hours from symptom onset to treatment, and limited reporting on long-term outcomes. Subgroup analyses including stratifying by diagnosis or treatment timing were not feasible due to small sample sizes and incomplete data. Also, a meta-analysis would add quantitative strength; however, due to the limited number of cases and the substantial heterogeneity in diagnoses, treatment regimens, outcome assessment, and reporting formats, a meta-analysis was not feasible at this stage. The primary goal of our review was not to establish efficacy but rather to aggregate existing evidence to motivate more rigorous future research into PGE1 application in ischemic conditions of the eye posterior segment. Therefore, we emphasize the urgent need for well-designed studies, such as multi-center randomized controlled trials to explore PGE1 as a potential therapeutic option for ocular ischemia. A potential standardized protocol for future trials may include intravenous PGE1 administered at a dose of 40 µg twice daily for 5 days, resulting in a cumulative dose of 400 µg. This regimen was the most frequently employed among the reviewed cases and appeared well tolerated without major adverse events. Its selection for clinical trial testing is further supported by its use in the largest patient cohorts.

**5.0 Conclusions**

Prostaglandin E1 appears as a potentially effective therapy for managing ischemic retinal and optic nerve diseases. The current evidence underscores the necessity for conducting extensive, controlled trials to evaluate the efficacy and safety profile of PGE1. Such research is crucial to substantiate its therapeutic potential and to integrate it into clinical practice for the treatment of various posterior segment ischemic disorders.

**Acknowledgements and Financial Disclosure**

**Funding/Support**: The publication of this article was funded by Qatar National Library.

**Financial Disclosures**: No financial disclosures.

**Authorship**: All authors attest that they meet the current ICMJE criteria for authorship.

**Conflict of interests**: None.

**References**

1. Hayreh SS. Management of ischemic optic neuropathies. *Indian J Ophthalmol*. Mar-Apr 2011;59(2):123-36. doi:10.4103/0301-4738.77024

2. Atebara NH, Brown GC, Cater J. Efficacy of anterior chamber paracentesis and Carbogen in treating acute nonarteritic central retinal artery occlusion. *Ophthalmology*. Dec 1995;102(12):2029-34; discussion 2034-5. doi:10.1016/s0161-6420(95)30758-0

3. Fieß A, Cal Ö, Kehrein S, Halstenberg S, Frisch I, Steinhorst UH. Anterior chamber paracentesis after central retinal artery occlusion: a tenable therapy? *BMC Ophthalmol*. Mar 10 2014;14:28. doi:10.1186/1471-2415-14-28

4. Hadanny A, Maliar A, Fishlev G, et al. Reversibility of retinal ischemia due to central retinal artery occlusion by hyperbaric oxygen. *Clin Ophthalmol*. 2017;11:115-125. doi:10.2147/opth.S121307

5. Powers WJ, Rabinstein AA, Ackerson T, et al. Guidelines for the Early Management of Patients With Acute Ischemic Stroke: 2019 Update to the 2018 Guidelines for the Early Management of Acute Ischemic Stroke: A Guideline for Healthcare Professionals From the American Heart Association/American Stroke Association. *Stroke*. Dec 2019;50(12):e344-e418. doi:10.1161/str.0000000000000211

6. Asdaghi N, Romano JG, Gardener H, et al. Thrombolysis in Mild Stroke. *Stroke*. 2021;52(10):e586-e589. doi:doi:10.1161/STROKEAHA.120.033466

7. Hakim N, Hakim J. Intra-Arterial Thrombolysis for Central Retinal Artery Occlusion. *Clin Ophthalmol*. 2019;13:2489-2509. doi:10.2147/opth.S232560

8. Hanchanale V, Eardley I. Alprostadil for the treatment of impotence. *Expert Opin Pharmacother*. Feb 2014;15(3):421-8. doi:10.1517/14656566.2014.873789

9. Singh Y, Mikrou P. Use of prostaglandins in duct-dependent congenital heart conditions. *Arch Dis Child Educ Pract Ed*. Jun 2018;103(3):137-140. doi:10.1136/archdischild-2017-313654

10. Malbin B, Padidam S, Burke M, et al. Intravenous Prostaglandin E1 Infusion for Acute Central Retinal Artery Occlusion. *Ophthalmic Surg Lasers Imaging Retina*. May 1 2019;50(5):S5-s8. doi:10.3928/23258160-20190108-02

11. Suzuki T, Obata R, Inoue T, et al. Intravenous lipo-prostaglandin E1 administration for patients with acute central retinal artery occlusion. *BMJ Open Ophthalmol*. May 2022;7(1)doi:10.1136/bmjophth-2022-001014

12. Steigerwalt RD, Jr., Belcaro G, Cesarone MR, et al. Branch retinal arterial occlusion treated with intravenous prostaglandin e1 and steroids. *Retin Cases Brief Rep*. Fall 2011;5(4):355-7. doi:10.1097/ICB.0b013e3182051de9

13. Ikeda N, Hayasaka S, Hayasaka Y, Murayama N. Poor vision for 2 days' duration and rapid visual recovery after treatment in a patient with branch retinal artery occlusion. *Annals of Ophthalmology*. 2004/03/01 2004;36(1):44-46. doi:10.1385/AO:36:1:44

14. Chacko JA, Broyles HV, Chacko JG, Uwaydat SH. Documented Reperfusion of the Retina on Fluorescein Angiography after Administration of Intravenous Prostaglandin E1 for Central Retinal Artery Occlusion: A Case Report. *Case Rep Ophthalmol*. Jan-Dec 2023;14(1):469-476. doi:10.1159/000533404

15. Takai Y, Tanito M, Matsuoka Y, Hara K, Ohira A. Systemic prostaglandin E1 to treat acute central retinal artery occlusion. *Invest Ophthalmol Vis Sci*. Apr 30 2013;54(4):3065-71. doi:10.1167/iovs.12-11445

16. Ohno Y, Kawai M, Arii Y, Mizutani S. Effect of prostaglandin E1 on ophthalmic artery velocimetry in a pre-eclamptic woman with visual disturbance caused by retinal arterial narrowing. *Gynecol Obstet Invest*. 2002;53(1):68-70. doi:10.1159/000049415

17. Steigerwalt RD, Jr., Pescosolido N, Corsi M, Cesarone MR, Belcaro GV. Acute branch retinal arterial embolism successfully treated with intravenous prostaglandin E1--case reports. *Angiology*. Jul-Aug 2003;54(4):491-3. doi:10.1177/000331970305400415

18. Muka T, Glisic M, Milic J, et al. A 24-step guide on how to design, conduct, and successfully publish a systematic review and meta-analysis in medical research. *Eur J Epidemiol*. Jan 2020;35(1):49-60. doi:10.1007/s10654-019-00576-5

19. Amir-Behghadami M, Janati A. Population, Intervention, Comparison, Outcomes and Study (PICOS) design as a framework to formulate eligibility criteria in systematic reviews. *Emerg Med J*. Jun 2020;37(6):387. doi:10.1136/emermed-2020-209567

20. M. Z. Moola S TC, Aromataris E, Sears K, Sfetcu R, Currie M, Qureshi R, Mattis P, Lisy K, Mu P-F. ***Systematic reviews of etiology and risk***. Joanna Briggs Institute Reviewer's Manual; 2017.

21. Steigerwalt RDJ, Nebbioso M. Visual improvement in a patient with paracentral acute middle maculopathy treated with prostaglandin E1. *Drug Discov Ther*. 2020;14(2):98-99. doi:10.5582/ddt.2020.03002

22. Steigerwalt RD, Jr., Pascarella A, De Angelis M, Grimaldi G, Nebbioso M. Three episodes of non-arteritic posterior ischemic optic neuropathy in the same patient treated with intravenous prostaglandin E1. *Drug Discov Ther*. 2016;10(3):177-80. doi:10.5582/ddt.2016.01036

23. Steigerwalt RDJ, Limoli PG, Nebbioso M. Visual field improvement in non-arteritic posterior ischemic optic neuropathy in a patient treated with intravenous prostaglandin E1 and steroids. *Drug Discov Ther*. 2017;11(4):226-229. doi:10.5582/ddt.2017.01034

24. Steigerwalt RD, Cesarone MR, Belcaro G, De Angelis M, Pascarella A, Nebbioso M. Non-arteritic Posterior Ischaemic Optic Neuropathy Treated with Intravenous Prostaglandin E1 and Oral Corticosteroids. *Neuroophthalmology*. 2011;35(2):81-84. doi:10.3109/01658107.2011.559564

25. Steigerwalt RD, Jr., Cesarone MR, Belcaro G, et al. Arteritic anterior ischemic optic neuropathy treated with intravenous prostaglandin E(1) and steroids. *Int J Angiol*. Fall 2010;19(3):e113-5. doi:10.1055/s-0031-1278380

26. Steigerwalt RD, Jr., Cesarone MR, Belcaro G, et al. Ocular ischemia in high myopia treated with intravenous prostaglandin e1. *Retin Cases Brief Rep*. Fall 2009;3(4):379-82. doi:10.1097/ICB.0b013e31817f2c80

27. Hayreh SS. Posterior ischaemic optic neuropathy: clinical features, pathogenesis, and management. *Eye (Lond)*. Nov 2004;18(11):1188-206. doi:10.1038/sj.eye.6701562

28. Steigerwalt RD, Jr., Belcaro GV, Christopoulos V, Incandela L, Cesarone MR, De Sanctis MT. Ocular and orbital blood flow velocity in patients with peripheral vascular disease and diabetes treated with intravenous prostaglandin E1. *J Ocul Pharmacol Ther*. Dec 2001;17(6):529-35. doi:10.1089/10807680152729211

29. Sobol EK, Sakai Y, Wheelwright D, et al. Intra-Arterial Tissue Plasminogen Activator for Central Retinal Artery Occlusion. *Clin Ophthalmol*. 2021;15:601-608. doi:10.2147/opth.S272126

30. Schrag M, Youn T, Schindler J, Kirshner H, Greer D. Intravenous Fibrinolytic Therapy in Central Retinal Artery Occlusion: A Patient-Level Meta-analysis. *JAMA Neurol*. Oct 2015;72(10):1148-54. doi:10.1001/jamaneurol.2015.1578

31. Simmet T, Peskar BA, Wolf HRD. On the Metabolism of Prostaglandin E1 in Patients Suffering from Arterial Occlusive Disease. Springer Berlin Heidelberg; 1986:8-12.

32. Lefer AM, Ogletree ML, Smith JB, et al. Prostacyclin: a potentially valuable agent for preserving myocardial tissue in acute myocardial ischemia. *Science*. Apr 7 1978;200(4337):52-4. doi:10.1126/science.345441

33. Hayreh SS, Zimmerman MB. Central retinal artery occlusion: visual outcome. *Am J Ophthalmol*. Sep 2005;140(3):376-91. doi:10.1016/j.ajo.2005.03.038

**Figure legends:**

**Figure 1.** A PRISMA flow diagram showing the database search and screening results.

**Table 1**. The baseline characteristics of included studies and subjects.

| **Study** | **Country** | **Study Design** | **Case** | **Age** | **Gender** | **Ethnicity** |
| --- | --- | --- | --- | --- | --- | --- |
| Ikeda et al ,2004^13^ | Japan | Case report | Case 1 | 82 | Male | - |
| Steigerwalt et al ,2011^12^ | Italy | Case report | Case 1 | 63 | Male | - |
| Steigerwalt et al ,2003^17^ | Italy | Case report | Case 1 | 82 | Female | - |
| Steigerwalt et al ,2017^23^ | Italy | Case report | Case 1 | 89 | Female | White |
| Steigerwalt et al ,2011^24^ | Italy | Case report | Case 1 | 68 | Male | White |
| Steigerwalt et al ,2016^22^ | Italy | Case report | Case 1 | 68 | Male | White |
| Steigerwalt et al,2020^21^ | Italy | Case report | Case 1 | 78 | Female | White |
| Chacko et al ,2023^14^ | USA | Case series | Case 1 | 67 | Male | Caucasian |
|  |  |  | Case 2 | 58 | Male | Black |
| Malbin et al ,2019^10^ | USA | Case series | Case 1 | 66 | Male | African American |
|  |  |  | Case 2 | 68 | Male | African American |
|  |  |  | Case 3 | 88 | Female | African American |
|  |  |  | Case 4 | 45 | Male | African American |
|  |  |  | Case 5 | 61 | Female | African American |
|  |  |  | Case 6 | 88 | Female | Caucasian |
| Takai et al ,2013^15^ | Japan | Case series | Case 1 | 52 | Female | - |
|  |  |  | Case 2 | 53 | Female | - |
|  |  |  | Case 3 | 48 | Male | - |
|  |  |  | Case 4 | 51 | Male | - |
|  |  |  | Case 5 | 59 | Female | - |
|  |  |  | Case 6 | 64 | Male | - |
|  |  |  | Case 7 | 56 | Male | - |
|  |  |  | Case 8 | 77 | Female | - |
|  |  |  | Case 9 | 74 | Female | - |
|  |  |  | Case 10 | 97 | Female | - |
| Steigerwalt et al ,2010^25^ | Italy | Case series | Case 1 | 75 | Female | White |
|  |  |  | Case 2 | 84 | Female | White |
| Steigerwalt et al ,2009^26^ | Italy | Case series | Case 1 | 40 | Male | - |
|  |  |  | Case 2 | 52 | Male | - |
|  |  |  | Case 3 | 68 | Female | - |

**Table 2**. The clinical characteristics of included subjects.

| **Study** | **Case** | **Affected eye side** | **diagnosis** | **Time from symptoms onset to presentation (h)** | **Ocular history** | **General History** |
| --- | --- | --- | --- | --- | --- | --- |
| Ikeda et al ,2004^13^ | Case 1 | left | BRAO | 2 | Extracapsular cataract | Aortic stenosis |
| Steigerwalt et al ,2011^12^ | Case 1 | right | BRAO | 3 | - | smoking |
| Steigerwalt et al ,2003^17^ | Case 1 | left | BRAO | 24 | Mild cataract in right eye | - |
| Steigerwalt et al ,2017^23^ | Case 1 | right | NA-PION | 66 | ocular surgery and macular degeneration | - |
| Steigerwalt et al ,2011^24^ | Case 1 | left | NA-PION | 24 | amblyopia in the right eye | hereditary haemochromatosis |
| Steigerwalt et al ,2016^22^ | Case 1 | left | NA-PION | 4 | amblyopia in the right eye | hereditary haemochromatosis |
| Steigerwalt et al,2020^21^ | Case 1 | left | PAMM | - | ocular hypertension | anemia |
| Chacko et al ,2023^14^ | Case 1 | left | CRAO | 6 | - | Hypertension, diabetes, CHF, atrial fibrillation and hyperlipidemia |
|  | Case 2 | left | CRAO | 12 | cataracts in both eyes | Smoking and hypertension |
| Malbin et al ,2019^10^ | Case 1 | left | CRAO | 12 | - | - |
|  | Case 2 | left | CRAO | 8 | - | - |
|  | Case 3 | right | CRAO | 2 | - | - |
|  | Case 4 | right | CRAO | 8 | - | - |
|  | Case 5 | left | CRAO | 12 | - | - |
|  | Case 6 | left | CRAO | 8 | - | - |
| Takai et al,2013^15^ | Case 1 | right | CRAO | 6 | Diabetic retinopathy | Hypertension and diabetes |
|  | Case 2 | left | CRAO | 2 | Diabetic retinopathy | Hypertension and diabetes |
|  | Case 3 | left | CRAO | 1 | - | Hypertension and diabetes |
|  | Case 4 | left | CRAO | 4 | - | Hypertension |
|  | Case 5 | left | CRAO | 17 | - | Hypertension |
|  | Case 6 | right | CRAO | 3 | - | - |
|  | Case 7 | right | CRAO | 2 | - | Hypertension |
|  | Case 8 | right | CRAO | 18 | Glaucoma | Hypertension |
|  | Case 9 | right | CRAO | 11 | - | Hypertension |
|  | Case 10 | right | CRAO | 7 | - | Hypertension |
| Steigerwalt et al ,2010^25^ | Case 1 | right | A-PION | - | - | general muscle pain, jaw claudication,  right-sided temporal pain, Diabetes and hypertension |
|  | Case 2 | left | A-PION | - | - | Diabetes, jaw claudication and left-sided temporal pain |
| Steigerwalt et al ,2009^26^ | Case 1 | left | ischemia in high myopia | 48 | cataracts removed, retinal detachment surgery and ocular hypertension | - |
|  | Case 2 | left | ischemia in high myopia | - | retinal detachment surgery in both eyes and macular  degeneration | smoking |
|  | Case 3 | left | ischemia in high myopia | - | cataract surgery in both eyes and macular degeneration | - |

**Table 3**. The characteristics of PGE1 treatment.

| **Study** | **Case** | **Initial dose (μg)** | **Frequency per day** | **Duration of treatment (days)** | **Cumulative dose until VA2 recording (μg)** |
| --- | --- | --- | --- | --- | --- |
| Ikeda et al ,2004^13^ | Case 1 | 60 | 1 | 1 | 60 |
| Steigerwalt et al ,2011^12^ | Case 1 | 80 | 1 | 2 | 160 |
| Steigerwalt et al ,2003^17^ | Case 1 | 140 | 1 | 2 | 280 |
| Steigerwalt et al ,2017^23^ | Case 1 | 60 | 1 | 2 | 120 |
| Steigerwalt et al ,2011^24^ | Case 1 | 60 | 1 | 2 | 120 |
| Steigerwalt et al ,2016^22^ | Case 1 | 60 | 1 | 2 | 120 |
| Steigerwalt et al,2020^21^ | Case 1 | 70 | 1 | 2 | 140 |
| Chacko et al ,2023^14^ | Case 1 | 40 | 2 | 1 | 80 |
|  | Case 2 | 40 | 2 | 2 | 160 |
| Malbin et al ,2019^10^ | Case 1 | 40 | 2 | 4 | 320 |
|  | Case 2 | 40 | 2 | 4 | 320 |
|  | Case 3 | 40 | 2 | 4 | 320 |
|  | Case 4 | 40 | 2 | 4 | 320 |
|  | Case 5 | 40 | 2 | 4 | 320 |
|  | Case 6 | 40 | 2 | 4 | 320 |
| Takai et al ,2013^15^ | Case 1 | 40 | 2 | 5 | 400 |
|  | Case 2 | 40 | 2 | 5 | 400 |
|  | Case 3 | 40 | 2 | 5 | 400 |
|  | Case 4 | 40 | 2 | 5 | 400 |
|  | Case 5 | 40 | 2 | 5 | 400 |
|  | Case 6 | 40 | 2 | 5 | 400 |
|  | Case 7 | 40 | 2 | 5 | 400 |
|  | Case 8 | 40 | 2 | 5 | 400 |
|  | Case 9 | 40 | 2 | 5 | 400 |
|  | Case 10 | 40 | 2 | 5 | 400 |
| Steigerwalt et al ,2010^25^ | Case 1 | 80 | 1 | 2 | 160 |
|  | Case 2 | 80 | 1 | 2 | 160 |
| Steigerwalt et al ,2009^26^ | Case 1 | 90 | 1 | 2 | 180 |
|  | Case 2 | 110 | 1 | 2 | 220 |
|  | Case 3 | 240 | 80 | 1 | 3 |

**Table 4**. The pre- and post-visual acuity of PGE1 treatment.

| **Study** | **Case** | **Visual acuity just before treatment initiation (VA1)** | **Visual acuity after treatment (VA2)** | **Visual acuity change (VA2 - VA1)** | **Time from treatment initiation until VA2 recording (days)** |
| --- | --- | --- | --- | --- | --- |
| Ikeda et al ,2004^13^ | Case 1 | 1.5 | 1 | -0.5 | 1 |
| Steigerwalt et al ,2011^12^ | Case 1 | 1 | 0.1 | -0.9 | 2 |
| Steigerwalt et al ,2003^17^ | Case 1 | 0.4 | 0 | -0.4 | 4 |
| Steigerwalt et al ,2017^23^ | Case 1 | 1 | 0.1 | -0.9 | 5 |
| Steigerwalt et al ,2011^24^ | Case 1 | 0.4 | 0 | -0.4 | 1 |
| Steigerwalt et al ,2016^22^ | Case 1 | 0.4 | 0 | -0.4 | 2 |
| Steigerwalt et al,2020^21^ | Case 1 | 1 | 0 | -1 | 9 |
| Chacko et al ,2023^14^ | Case 1 | 2.7 | 2.3 | -0.4 | 1 |
|  | Case 2 | 3 | 1.8 | -1.2 | 2 |
| Malbin et al ,2019^10^ | Case 1 | 2.7 | 2.3 | -0.4 | 7 |
|  | Case 2 | 3 | 2.7 | -0.3 | 7 |
|  | Case 3 | 2.7 | 2.3 | -0.4 | 7 |
|  | Case 4 | 2.3 | 0.4 | -1.9 | 7 |
|  | Case 5 | 2.7 | 1.3 | -1.4 | 7 |
|  | Case 6 | 3 | 2.7 | -0.3 | 7 |
| Takai et al ,2013^15^ | Case 1 | 3 | 0.3 | -2.7 | 30 |
|  | Case 2 | 1.7 | 0.2 | -1.5 | 30 |
|  | Case 3 | 3 | 1.5 | -1.5 | 30 |
|  | Case 4 | 3 | 1 | -2 | 30 |
|  | Case 5 | 2 | 0.7 | -1.3 | 30 |
|  | Case 6 | 3 | 0.4 | -2.6 | 30 |
|  | Case 7 | 3 | 0 | -3 | 30 |
|  | Case 8 | 3 | 2 | -1 | 30 |
|  | Case 9 | 3 | 0.2 | -2.8 | 30 |
|  | Case 10 | 2 | 0.5 | -1.5 | 30 |
| Steigerwalt et al ,2010a^25^ | Case 1 | 1.1 | 0.3 | -0.8 | 2 |
|  | Case 2 | 1.3 | 1 | -0.3 | 7 |
| Steigerwalt et al ,2009^26^ | Case 1 | 2.3 | 1 | -1.3 | 3 |
|  | Case 2 | 0.7 | 0.2 | -0.5 | 2 |
|  | Case 3 | 2.3 | 0.6 | -1.7 | 3 |

**Table 5**. The overall patients’ characteristics.

| **Patients’ characteristics** |  | **Overall** |
| --- | --- | --- |
| Age, (mean (SD)) |  | 66.77 (13.63) |
| Gender (%) | female | 15 (50.0) |
|  | male | 15 (50.0) |
| Eye side (%) | left | 19 (63.3) |
|  | right | 11 (36.7) |
| Diagnosis (%) | A-AION | 1 ( 3.3) |
|  | A-PION | 1 ( 3.3) |
|  | BRAO | 3 (10.0) |
|  | CRAO | 18 (60.0) |
|  | ischemia in high myopia | 3 (10.0) |
|  | NA-PION | 3 (10.0) |
|  | PAMM | 1 ( 3.3) |
| Time from symptoms onset to treatment initiation (hours), (median [IQR]) |  | 8.00 [3.00, 12.00] |
| Initial IV PGE1 dose (μg), (median [IQR]) |  | 40.00 [40.00, 60.00] |
| Frequency of dose per day, (median [IQR]) |  | 2.00 [1.00, 2.00] |
| Duration of treatment with IV PGE1 (days), (median [IQR]) |  | 4.00 [2.00, 5.00] |
| Cumulative dose until VA2 recording (μg),  (median [IQR]) |  | 320.00 [160.00, 400.00] |
| Time from treatment initiation to VA2 recording (days), (median [IQR]) |  | 7.00 [2.25, 30.00] |
| VA1 (logMAR), (median [IQR]) |  | 2.30 [1.15, 3.00] |
| VA2 (logMAR), (median [IQR]) |  | 0.55 [0.20, 1.45] |
| Change in Visual acuiy (logMAR),  (median [IQR]) |  | -1.00 [-1.50, -0.40] |
| Steroids (%) | Not used | 26 (86.7) |
|  | used | 4 (13.3) |
